# Supplementary material for: Time-series transcriptome analysis identified differentially expressed genes in broiler chicken infected with mixed Eimeria species
Source: Front Genet. 2022 Aug 8;13:886781. doi: 10.3389/fgene.2022.886781 (PMC9393255; doi:10.3389/fgene.2022.886781)
Supplement: Supplementary file 2 [file DataSheet1.ZIP › 4dpi_KEGG.Gsea.1625100298584/KEGG_SULFUR_METABOLISM.html]

Details for gene set KEGG\_SULFUR\_METABOLISM[GSEA]

|  || Dataset | TMM\_4dpi\_gct\_format\_4dpi\_gct\_format.Class\_4dpi.cls #PC\_versus\_NC.Class\_4dpi.cls #PC\_versus\_NC\_repos |
| Phenotype | Class\_4dpi.cls#PC\_versus\_NC\_repos |
| Upregulated in class | 0 |
| GeneSet | KEGG\_SULFUR\_METABOLISM |
| Enrichment Score (ES) | -0.6423112 |
| Normalized Enrichment Score (NES) | -1.3251097 |
| Nominal p-value | 0.15235342 |
| FDR q-value | 0.4116985 |
| FWER p-Value | 0.9998 |
Table: GSEA Results Summary

  

Fig 1: Enrichment plot: KEGG\_SULFUR\_METABOLISM      
 Profile of the Running ES Score & Positions of GeneSet Members on the Rank Ordered List

  

| SYMBOL | TITLE | RANK IN GENE LIST | RANK METRIC SCORE | RUNNING ES | CORE ENRICHMENT || 1 | CHST12 | na | 1231 | 0.594 | 0.0429 | No |
| 2 | PAPSS2 | na | 2558 | 0.341 | 0.0159 | No |
| 3 | CHST13 | na | 5352 | 0.050 | -0.2046 | No |
| 4 | PAPSS1 | na | 10182 | -0.442 | -0.4986 | Yes |
| 5 | BPNT1 | na | 11908 | -1.314 | -0.3205 | Yes |
| 6 | SULT1E1 | na | 11912 | -1.343 | 0.0082 | Yes |
Table: GSEA details [plain text format]

  

Fig 2: KEGG\_SULFUR\_METABOLISM      
 Blue-Pink O' Gram in the Space of the Analyzed GeneSet

  

Fig 3: KEGG\_SULFUR\_METABOLISM: Random ES distribution      
 Gene set null distribution of ES for **KEGG\_SULFUR\_METABOLISM**
